# Supplementary material for: Cost-effort analysis of Baited Remote Underwater Video (BRUV) and environmental DNA (eDNA) in monitoring marine ecological communities
Source: PeerJ. 2024 Apr 30;12:e17091. doi: 10.7717/peerj.17091 (PMC11067900; doi:10.7717/peerj.17091)
Supplement: Supplemental Information 1 — GPS coordinates (Latitude and Longitude) and Easting and Northing of eDNA and BRUV sampling sites [file peerj-12-17091-s001.docx]

***Table 1: GPS coordinates and Easting and Northing of eDNA and BRUV sampling sites***

| **Site** | **Latitude (degrees, decimal, mins)** | **Longitude (degrees, decimal, mins)** | **Easting** | **Northing** |
| --- | --- | --- | --- | --- |
| 1 | 50 48.981 | 0 12.265 | 526588 | 103403 |
| 2 | 50 48.4 | 0 12.643 | 526170 | 102316 |
| 3 | 50 46.867 | 0 12.680 | 526280 | 99476 |
| 4 | 50 49.088 | 0 17.532 | 520400 | 103455 |
| 5 | 50 48.072 | 0 17.542 | 520432 | 101572 |
| 6 | 50 46.622 | 0 17.559 | 520474 | 98884 |
| 7 | 50 47.928 | 0 24.201 | 512617 | 101130 |
| 8 | 50 46.993 | 0 24.21 | 512644 | 99397 |
| 9 | 50 45.572 | 0 24.251 | 512652 | 96763 |
| 10 | 50 47.726 | 0 29.024 | 506960 | 100636 |
| 11 | 50 47.098 | 0 29.024 | 506984 | 99473 |
| 12 | 50 45.536 | 0 29.085 | 506971 | 96576 |
| 13 | 50 47.187 | 0 35.258 | 499657 | 99493 |
| 14 | 50 46.286 | 0 35.349 | 499582 | 97821 |
| 15 | 50 44.702 | 0 35.240 | 499766 | 94888 |
| 16 | 50 46.757 | 0 39.030 | 495240 | 98613 |
| 17 | 50 45.808 | 0 39.055 | 495242 | 96854 |
| 18 | 50 44.643 | 0 39.001 | 495345 | 94696 |
| 19 | 50 45.987 | 0 42.660 | 490999 | 97110 |
| 20 | 50 44.232 | 0 42.352 | 491418 | 93864 |
| 21 | 50 43.063 | 0 41.363 | 492620 | 91718 |
| 22 | 50 43.325 | 0 46.040 | 487109 | 92109 |
| 23 | 50 42.868 | 0 45.623 | 487614 | 91270 |
| 24 | 50 42.447 | 0 45.283 | 488027 | 90496 |
| 25 | 50 40.773 | 0 44.022 | 489564 | 87419 |
| 26 | 50 44.388 | 0 24.300 | 512642 | 94567 |
| 27 | 50 44.515 | 0 26.341 | 510237 | 94751 |
| 28 | 50 44.272 | 0 29.077 | 507029 | 94234 |
| Swanage | 50 35.605 | 1 57.136 | 526588 | 103403 |
