# Supplementary material for: Cost-effort analysis of Baited Remote Underwater Video (BRUV) and environmental DNA (eDNA) in monitoring marine ecological communities
Source: PeerJ. 2024 Apr 30;12:e17091. doi: 10.7717/peerj.17091 (PMC11067900; doi:10.7717/peerj.17091)
Supplement: Supplemental Information 2 — Overall average cost breakdown per year (for 5 years of sampling) of each biomonitoring method to sample 28 sites and cost incurred per species detected and per site sampled. [file peerj-12-17091-s002.docx]

***Table 2: Overall average cost breakdown per year (for 5 years of sampling) of each biomonitoring method to sample 28 sites and cost incurred per species detected and per site sampled.***

| **BRUV COSTS** | | **eDNA costs**  **(external analysis)** | | **eDNA costs**  **(in-house analysis)** | |
| --- | --- | --- | --- | --- | --- |
| 3 BRUV systems | £6,000 | eDNA filter kits x32 | £1,600 | eDNA filter kits x32 | £1,600 |
| 9 GoProHero8 cameras | £3,150 | Kemmerer sampler | £800 | Kemmerer sampler | £800 |
| Bait (Atlantic horse mackerel)  6 fish x 28 sites = 168 fish | £65 | Vampire suction drill rental | £112 | Vampire suction drill rental | £112 |
| Boat hire + fuel x 12 days | £4,500 | Boat hire + fuel x 12 days | £4,500 | Boat hire + fuel x 12 days | £4,500 |
| BRUV footage analysis (95hours x £10.9) | £1,035 | eDNA analysis | £7,700 | eDNA analysis | £5372 |
| **TOTAL YEAR 1** | **£14,750** | **TOTAL YEAR 1** | **£14,712** | **TOTAL YEAR 1** | **£12,385** |
| Years 2,3,4,5 (bait, boat, BRUV maintenance) | £5,600 p.a. | Years 2,3,4,5 (bait, boat, kits, analysis) | £13,912 p.a. | Years 2,3,4,5 (bait, boat, kits, analysis) | £11,585 |
| **AVERAGE over 5 years** | **£ 7,430 p.a.** | **AVERAGE over 5 years** | **£14,072 p.a.** | **AVERAGE over 5 years** | **£11,745** |
| **Average per site** | **£265** | **Average per site** | **£502** | **Average per site** | **£420** |
| **Average per species detected (26)** | **£285** | **Average per species detected (78)** | **£180** | **Average per species detected (78)** | **£150** |
