# Supplementary material for: Cost-effort analysis of Baited Remote Underwater Video (BRUV) and environmental DNA (eDNA) in monitoring marine ecological communities
Source: PeerJ. 2024 Apr 30;12:e17091. doi: 10.7717/peerj.17091 (PMC11067900; doi:10.7717/peerj.17091)
Supplement: Supplemental Information 3 — List of the species sampled by eDNA, BRUV and both methods. [file peerj-12-17091-s003.docx]

***Table 3: Species sampled by each method***

| BRUVs alone | | eDNA alone | | Both | |
| --- | --- | --- | --- | --- | --- |
| Scientific name | **Common name** | **Scientific name** | **Common name** | **Scientific name** | **Common name** |
| *Chirolophis ascanii* | Yarrells blenny | *Ammodytes spp.* | *Ammodytes spp.* | *Dicentrarchus labrax* | European seabass |
| *Chelidonichthys lucerna* | Tub gurnard | *Anguilla anguilla* | European eel | *Conger conger* | Conger eel |
| *Galeorhinus galeus* | Tope | *Aphia spp.* | *Aphia spp.* | *Gobius paganellus* | Rock goby |
|  |  | *Aphia minuta* | Transparent goby | *Ctenolabrus rupestris* | Goldsinny wrasse |
|  |  | *Arnoglossus laterna* | Scaldfish | *Gobiusculus flavescens* | Two-spot goby |
|  |  | *Atherina presbyter* | Sand smelt | *Scyliorhinus canicula* | Small spotted catshark |
|  |  | *Apletodon dentatus* | Small-headed clingfish | *Mustelus asterias* | Starry smooth-hound |
|  |  | *Atherina boyeri* | Big-scale sand smelt | *Raja undulata* | Undulate ray |
|  |  | *Buglossidium luteum* | Solonette | *Scomber scombrus* | Atlantic mackerel |
|  |  | *Belone belone* | Garfish | *Raja clavata* | Thornback ray |
|  |  | *Blennius ocellaris* | Butterfly blenny | *Pomatoschistus pictus* | Painted goby |
|  |  | *Callionymus spp.* | *Callionymus spp.* | *Sparus aurata* | Gilt-head seabream |
|  |  | *Callionymus lyra* | Common dragonet | *Spondyliosoma cantharus* | Black seabream |
|  |  | *Chelidonichthys lastoviza* | Streaked gurnard | *Symphodus melops* | Corkwing wrasse |
|  |  | *Ciliata mustela* | Fivebeard rockling | *Thorogobius ephippiatus* | Leopard-spotted goby |
|  |  | *Chelon auratus* | Golden grey mullet | *Trisopterus minutus* | Poor cod |
|  |  | *Ciliata septentrionalis* | Northern rockling | *Trisopterus luscus* | Pouting |
|  |  | *Cottus gobio* | European bullhead | *Mullus surmuletus* | Striped red mullet |
|  |  | *Clupea spp.* | *Clupea spp.* | *Labrus mixtus* | Cuckoo wrasse |
|  |  | *Clupea harengus* | Atlantic herring | *Labrus bergylta* | Ballan wrasse |
|  |  | *Diplecogaster bimaculata* | Two-spot clingfish | *Lipophrys pholis* | Shanny |
|  |  | *Echiichthys vipera* | Lesser weever | *Pollachius pollachius* | Pollock |
|  |  | *Dasyatis pastinaca* | Common stingray | *Pomatoschistus microps* | Common goby |
|  |  | *Engraulis encrasicolus* | European anchovy | *Pomatoschistus minutus* | Sand goby |
|  |  | *Gaidropsarus vulgaris* | Three-bearded rockling |  |  |
|  |  | *Gobius spp.* | *Gobius spp.* |  |  |
|  |  | *Gobius niger* | Black goby |  |  |
|  |  | *Hyperoplus lanceolatus* | Great sandeel |  |  |
|  |  | *Gasterosteus aculeatus* | Three-spined stickleback |  |  |
|  |  | *Liparis montagui* | Montagu's snailfish |  |  |
|  |  | *Chelon aurata* | Golden mullet |  |  |
|  |  | *Lepadogaster candollei* | Connemara clingfish |  |  |
|  |  | *Limanda limanda* | Common dab |  |  |
|  |  | *Merlangius merlangus* | Whiting |  |  |
|  |  | *Micrenophrys lilljeborgii* | Norway bullhead |  |  |
|  |  | *Nerophis lumbriciformis* | Worm pipefish |  |  |
|  |  | *Maurolicus muelleri* | Mueller's pearlside |  |  |
|  |  | *Pholis gunnellus* | Rock gunnel |  |  |
|  |  | *Pleuronectes platessa* | European plaice |  |  |
|  |  | *Pomatoschistus lozanoi* | Lozano's goby |  |  |
|  |  | *Pungitius pungitius* | Nine-spined stickleback |  |  |
|  |  | *Salmo salar* | Atlantic salmon |  |  |
|  |  | *Sardina pilchardus* | European pilchard |  |  |
|  |  | *Scophthalmus rhombus* | Brill |  |  |
|  |  | *Solea solea* | Dover sole |  |  |
|  |  | *Sprattus sprattus* | Sprat |  |  |
|  |  | *Psetta maxima* | European turbot |  |  |
|  |  | *Syngnathus typhle* | Broadnosed pipefish |  |  |
|  |  | *Symphodus bailloni* | Baillon's wrasse |  |  |
|  |  | *Syngnathus acus* | Greater pipefish |  |  |
|  |  | *Taurulus bubalis* | Long-spined sea scorpion |  |  |
|  |  | *Tursiops truncatus* | Common bottlenose |  |  |
|  |  | *Pegusa lascaris* | Sand sole |  |  |
|  |  | *Parablennius gattorugine* | Tompot blenny |  |  |
|  |  |  |  |  |  |
|  |  |  |  |  |  |
