# Supplementary figures and images for: Cost-effort analysis of Baited Remote Underwater Video (BRUV) and environmental DNA (eDNA) in monitoring marine ecological communities
Source: PeerJ. 2024 Apr 30;12:e17091. doi: 10.7717/peerj.17091 (PMC11067900; doi:10.7717/peerj.17091)

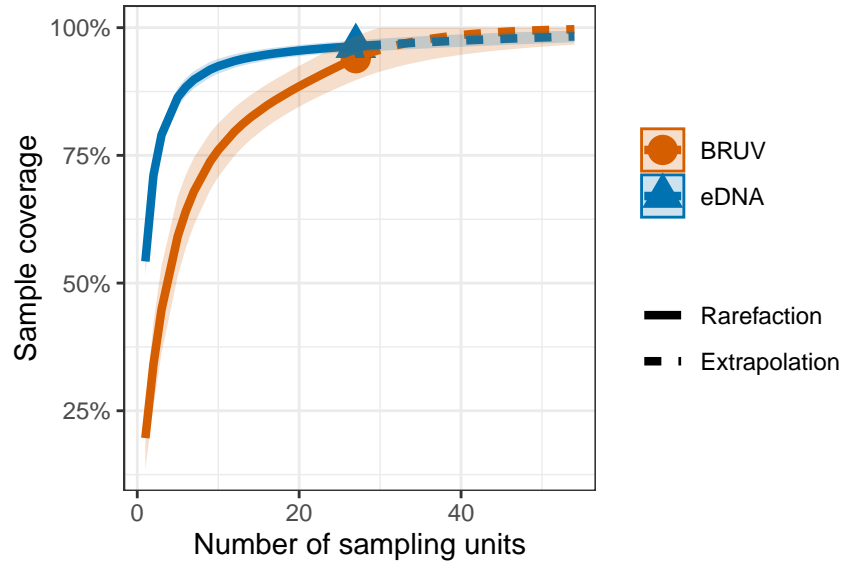

Supplement: Supplemental Information 4 — The coverage-based estimate of diversity (q = 0) for 21 samples was 93.7%, for 42 samples it was 96.5% and for 63 samples it was 98.4%. [file peerj-12-17091-s004.pdf]

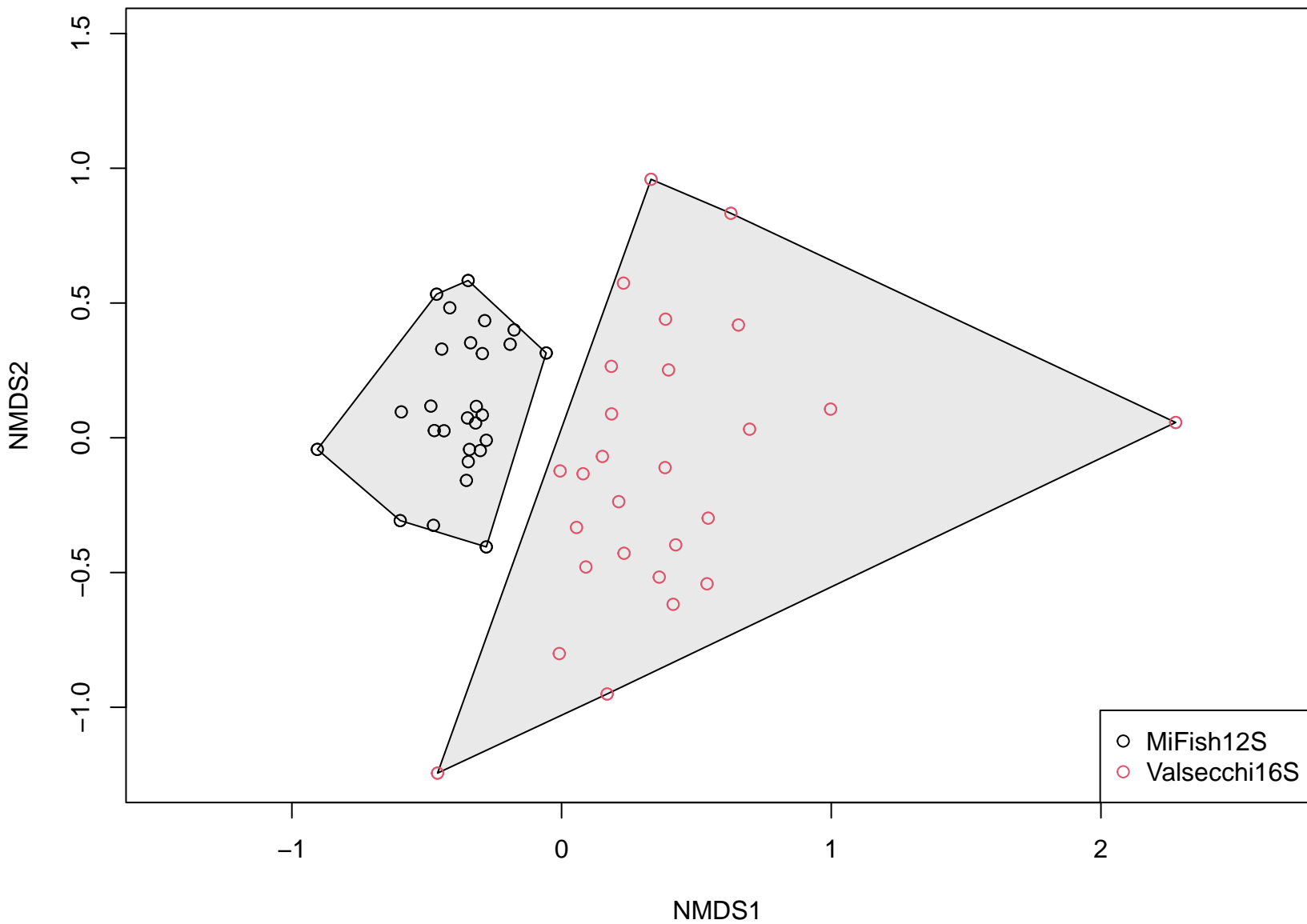

Supplement: Supplemental Information 5 — Non-Metric Multidimensional Scaling (nMDS) of Sussex sites showing community composition identified by MiFish 12S and Valsecchi 16S [file peerj-12-17091-s005.pdf]

mod

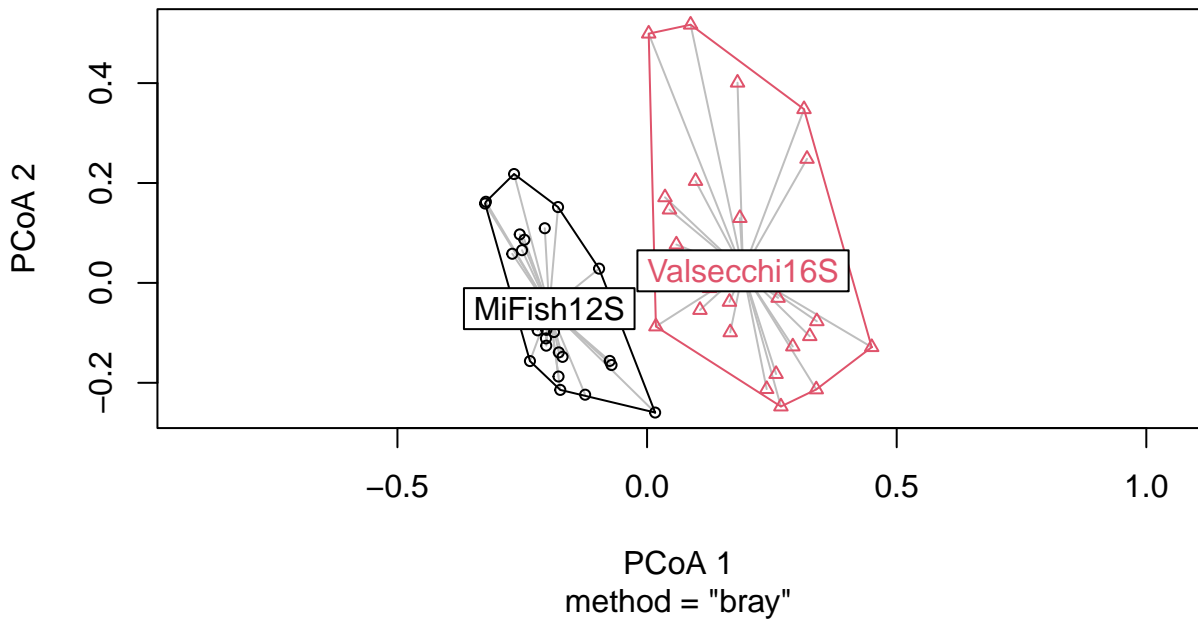

Supplement: Supplemental Information 6 — Principal Coordinates Analysis (PCoA) was conducted on Jaccard dissimilarities of fish assemblages of all species detected across eDNA surveys, grouped by primer (Valsecchi 16S and MiFish 12S). We found a significant difference in homogeneity of dispersions between primers (betadisper ¡ 0.05). [file peerj-12-17091-s006.pdf]

**mod**

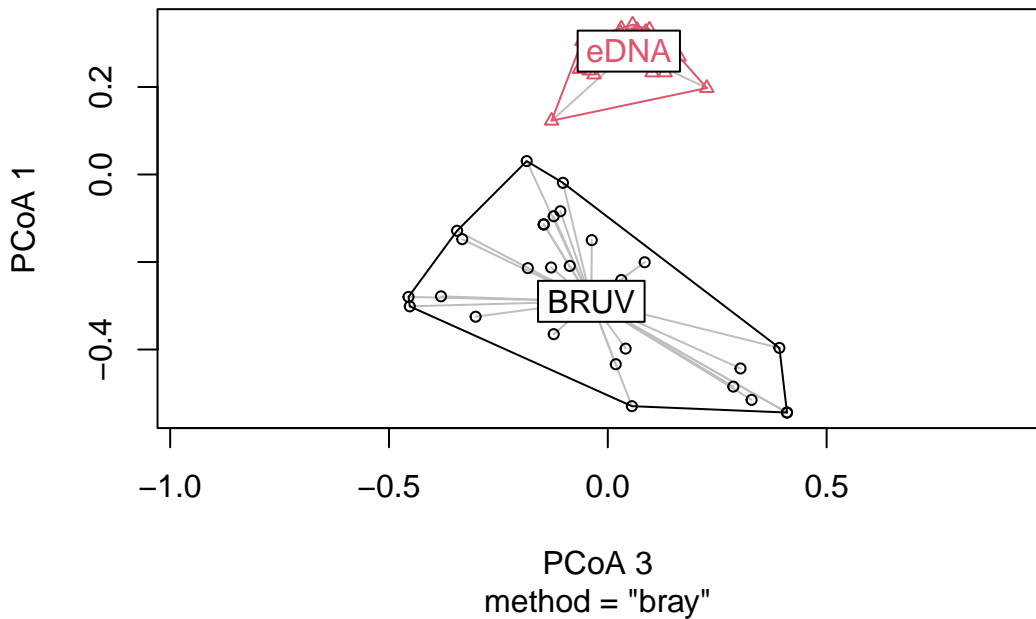

Supplement: Supplemental Information 7 — Principal Coordinates Analysis (PCoA) was conducted on Jaccard dissimilarities of fish assemblages of all species detected across eDNA surveys, grouped by method (eDNA and BRUV). We found a significant difference in homogeneity of dispersions between the two methods (betadisper ¡ 0.05). [file peerj-12-17091-s007.pdf]
